# Supplementary material for: The interactome of intact mitochondria by cross-linking mass spectrometry provides evidence for coexisting respiratory supercomplexes
Source: Mol Cell Proteomics. 2017 Dec 8;17(2):216–32. doi: 10.1074/mcp.RA117.000470 (PMC5795388; doi:10.1074/mcp.RA117.000470)
Supplement: Supplemental Data [file supp_RA117.000470_133922_0_supp_23978_fzffwf.zip › spectra_annotation/mito_spectra_annotation/90-1-9-1-20-1.pdf]

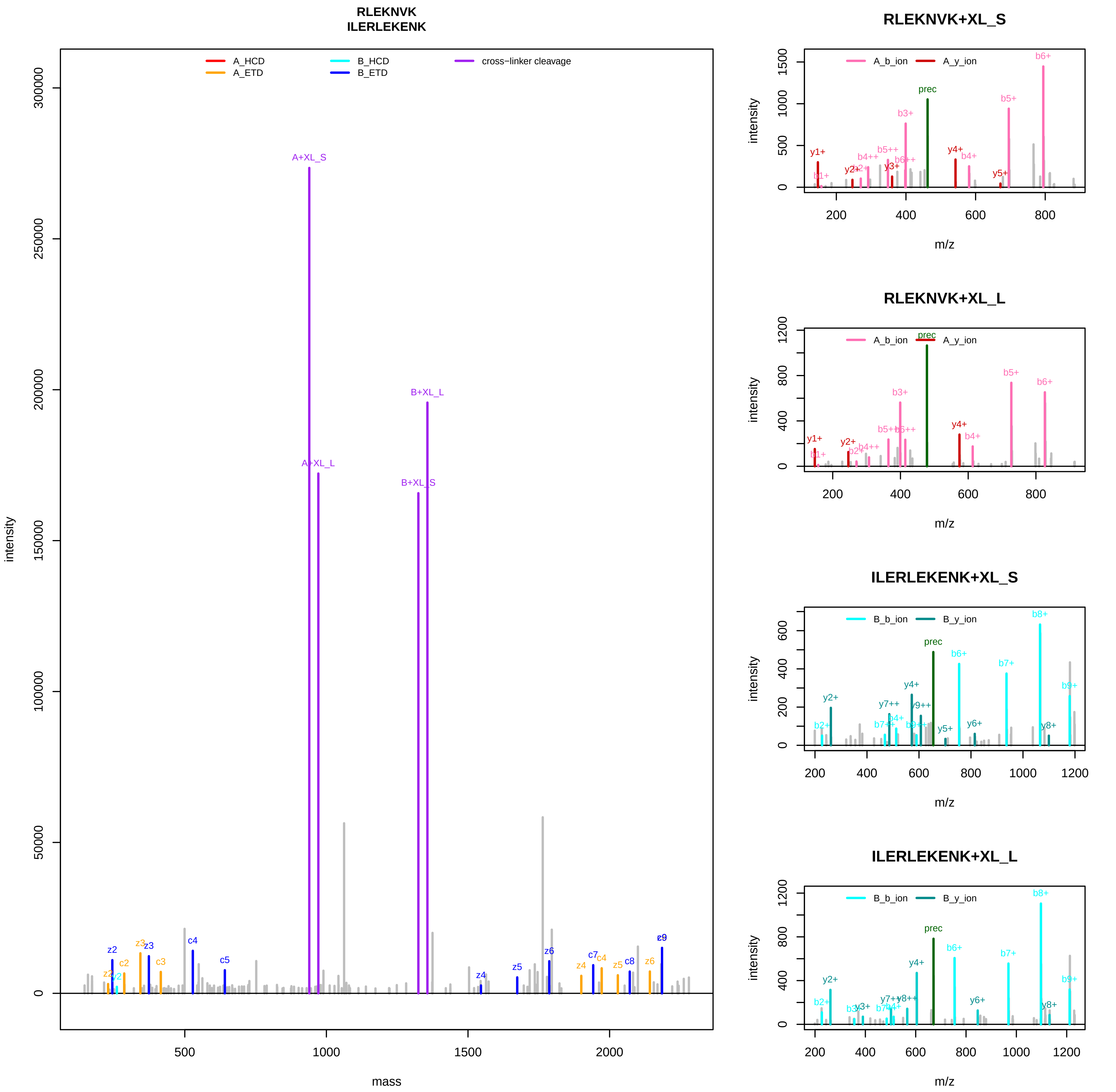
RLEKNVK
ILERLEKENK
A_HCD
A_ETD
B_HCD
B_ETD
cross−linker cleavage
A+XL_S
A+XL_L
B+XL_L
B+XL_S
z2 z2 y2 c2 z3 z3 c3 c4 c5 z4 z5 z6 z4 c7 c4 z5 c8 z6 c9
0 50000 100000 150000 200000 250000 300000
500 1000 1500 2000
intensity
mass
RLEKNVK+XL_S
A_b_ion
A_y_ion
y1+ b1+ y2+ b2+ b4++ b5++ y3+ b6++ b3+ prec y4+ b4+ y5+ b5+ b6+
0 500 1000 1500
200 400 600 800
intensity
m/z
RLEKNVK+XL_L
A_b_ion
A_y_ion
y1+ b1+ y2+ b2+ b4++ b5++ b6++ b3+ prec y4+ b4+ b5+ b6+
0 400 800 1200
200 400 600 800
intensity
m/z
ILERLEKENK+XL_S
B_b_ion
B_y_ion
b2+ y2+ b7++ y7++ b4+ y4+ y9++ b9++ prec y5+ b6+ y6+ b7+ b8+ y8+ b9+
0 200 400 600
200 400 600 800 1000 1200
intensity
m/z
ILERLEKENK+XL_L
B_b_ion
B_y_ion
b2+ y2+ b3+ y3+ b7++ y7++ b4+ y8++ y4+ prec b6+ y6+ b7+ b8+ y8+ b9+
0 400 800 1200
200 400 600 800 1000 1200
intensity
m/z
